# Optical-resolution parallel ultraviolet photoacoustic microscopy for slide-free histology

**DOI:** 10.1126/sciadv.ado0518

**Published:** 2024-12-11

**Authors:** Rui Cao, Yilin Luo, Jingjing Zhao, Yushun Zeng, Yide Zhang, Qifa Zhou, Adam le da Zerda, Lihong V. Wang

**Affiliations:** ^1^ Caltech Optical Imaging Laboratory, Andrew and Peggy Cherng Department of Medical Engineering, Department of Electrical Engineering, California Institute of Technology, Pasadena, CA 91125, USA.; ^2^ Department of Structural Biology, Stanford University, Stanford, CA 94305, USA.; ^3^ Alfred E. Mann Department of Biomedical Engineering, University of Southern California, Los Angeles, CA 90089, USA.; ^4^ Department of Ophthalmology, University of Southern California, Los Angeles, CA 90033, USA.; ^5^ Department of Electrical Engineering, Stanford University, Stanford, CA 94305, USA.; ^6^ The Chan Zuckerberg Biohub, San Francisco, CA 94158, USA.

## Abstract

Intraoperative imaging of slide-free specimens is crucial for oncology surgeries, allowing surgeons to quickly identify tumor margins for precise surgical guidance. While high-resolution ultraviolet photoacoustic microscopy has been demonstrated for slide-free histology, the imaging speed is insufficient, due to the low laser repetition rate and the limited depth of field. To address these challenges, we present parallel ultraviolet photoacoustic microscopy (PUV-PAM) with simultaneous scanning of eight optical foci to acquire histology-like images of slide-free fresh specimens, improving the ultraviolet PAM imaging speed limited by low laser repetition rates. The PUV-PAM has achieved an imaging speed of 0.4 square millimeters per second (i.e., 4.2 minutes per square centimeter) at 1.3-micrometer resolution using a 50-kilohertz laser. In addition, we demonstrated the PUV-PAM with eight needle-shaped beams for an extended depth of field, allowing fast imaging of slide-free tissues with irregular surfaces. We believe that the PUV-PAM approach will enable rapid intraoperative photoacoustic histology and provide prospects for ultrafast optical-resolution PAM.

## Teaser

Parallel ultraviolet photoacoustic microscopy with needle-shaped beams for rapid slide-free photoacoustic histology.

## INTRODUCTION

Fast and accurate histological analysis is essential in surgical oncology, playing a decisive role in influencing surgical decisions and directly determining patient outcomes. Extensive research has consistently demonstrated a definitive link between the completeness of tumor resection and enhanced survival rates in patients (*1*, *2*), highlighting the need for careful and precise decision-making during surgery to achieve the best results. However, current intraoperative pathology mostly relies on frozen section techniques, which encounter challenges in clinical practice. While invaluable for their immediacy, frozen section techniques are susceptible to inaccuracies due to freezing artifacts, leading to uncertainty in surgical decision-making (*3*). In addition, the utility of frozen sections is particularly limited in certain tissues such as bone and fat tissues, leading to challenges in their use across various surgical contexts. Furthermore, the process of preparing frozen sections often results in unnecessary tissue loss, which may affect the subsequent pathological analysis.

The limitations inherent in traditional histological methods underscore the critical demand for innovative imaging technologies in the field of surgical oncology (*4*). Recent developments in imaging techniques, such as microscopy with ultraviolet surface excitation (MUSE) (*5*, *6*), light sheet microscopy (*7*–*9*), optical coherence tomography (OCT) (*10*, *11*), and stimulated Raman scattering (SRS) microscopy (*12*, *13*), offer promising alternatives. Nevertheless, each of these advanced modalities comes with its own challenges, which may limit their effectiveness in intraoperative settings. MUSE, known for its high-resolution surface imaging capabilities within minutes, necessitates the dye staining of specimens to achieve histological contrast (*5*, *6*). This process varies across different tissue types and requires the expertise for dye staining of each tissue type. Light sheet microscopy, while advantageous for its rapid imaging capabilities and reduced phototoxicity, faces limitations due to its reliance on optical transparency (*9*). OCT excels in providing real-time, cross-sectional imaging, yet it falls short in molecular specificity and is unable to offer direct nuclear contrast within tissues, a limitation stemming from inadequate chromophore specificity of its optical scattering contrast (*11*). SRS microscopy, notable for its label-free imaging and high molecular specificity, is impeded by comparatively slow imaging speeds, making it less ideal for time-sensitive surgical procedures (*12*).

By detecting the optical absorption-induced acoustic signals, photoacoustic microscopy (PAM) can image the distribution of intrinsic or extrinsic optical absorbers label free (*14*, *15*). In recent years, PAM has been widely used to image various biological contrasts, including hemoglobin (*16*), DNA/RNA (*17*, *18*), cytochrome (*19*), melanoma (*20*), and lipid (*21*, *22*). With a tightly focused beam spot, the optical-resolution PAM (OR-PAM) can achieve the optical diffraction-limited resolution. Through scanning of either the optical focus or the sample, OR-PAM two-dimensional (2D) maximal amplitude projection images or 3D volumetric images can be reconstructed using photoacoustic signals and their time-of-flight information. The resulting OR-PAM images provide information on tissue morphology, functional properties, and molecular composition, enabling the study of biological processes at the cellular and subcellular level.

With strong absorption of nuclei and protein at the ultraviolet wavelength range, ultraviolet PAM (UV-PAM) has emerged as a powerful tool for fast, label-free histological imaging of tissues like bone and breast tissue (*17*, *23*). However, the imaging speed of UV-PAM has been limited by the low pulse repetition rate (PRR) of conventional ultraviolet pulsed lasers. To mitigate this, Martell *et al.* (*24*) have developed a customized ultraviolet laser to improve imaging speed of UV-PAM, using the second harmonic of a 532-nm pulsed laser at a PRR of 10 to 2000 kHz and a cesium lithium borate crystal to generate 266-nm laser pulses. Yet, such customized lasers are challenging to maintain and not readily accessible. Another strategy to increase imaging speed is through parallel imaging with an ultrasonic transducer array and a microlens array (*25*). However, this approach, often constrained to transmission mode PAM, is not suitable for imaging slide-free specimens due to its bulky configuration and short working distance of the microlens array for high-resolution imaging. Moreover, the requirement for transducer arrays and multichannel high-speed data acquisition systems substantially increases the system cost.

In this work, we report a parallel UV-PAM (PUV-PAM) in reflection mode via customized diffractive optical elements (DOEs) and two single-element ultrasonic transducers, achieving slide-free histology-like imaging at 400-kHz pixel rate using a 266-nm pulsed laser with a PRR of 50 kHz. The parallel scanning was implemented with a hybrid scanning method, consisting of galvo scanning of eight focal spots generated via a diffractive beam splitter and motor scanning of the specimen. With this reflection-mode PUV-PAM, we can achieve the imaging speed of 0.4 mm^2^/s (i.e., 4.2 min/cm^2^) at the resolution of 1.3 μm. To overcome the challenge by the irregular surface of thick slide-free tissues, we also demonstrated parallel needle-shaped beams in the PUV-PAM with an extended depth of field via a customized DOE, enabling fast intraoperative slide-free photoacoustic histology of various tissues with irregular surfaces. We believe that this system will shed light on the future development of fast PAM and have the potential to revolutionize slide-free photoacoustic histology.

## RESULTS

### Reflection-mode PUV-PAM with dual ultrasonic transducers

**Fig. 1.**
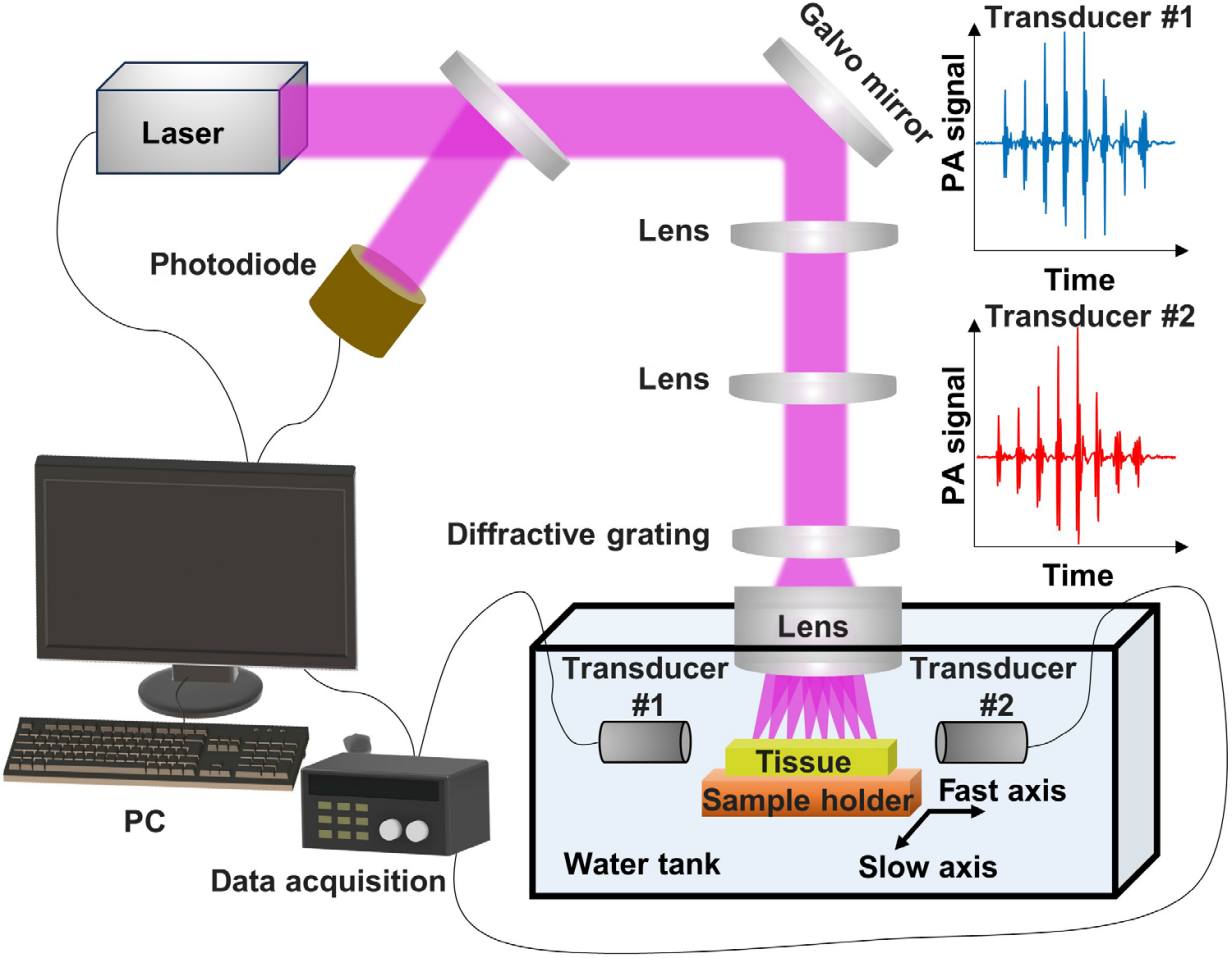
Schematic of optical-resolution PUV-PAM in reflection mode. Experimental setup of the optical-resolution PUV-PAM and representative photoacoustic (PA) signals from two ultrasonic transducers.

Our optical-resolution PUV-PAM system uses a 50-kHz PRR laser at 266 nm, generating strong photoacoustic signals from cellular components like cell nuclei and cytoplasm for histology-like images (Fig. 1). A customized ultraviolet F-theta objective lens and a diffractive grating for beam splitting are used to create eight focused spots at a spacing of 375 μm. One advantage of using a diffractive grating alongside an objective lens, as opposed to a microlens array, is achieving a larger working distance while maintaining a high numerical aperture (NA), thanks to the large beam diameter of the split beams. The customized ultraviolet F-theta objective lens is designed to effectively minimize field curvature with the NA of ~0.15 (fig. S1A). The use of the F-theta objective lens and the diffractive grating ensures linear scanning at the focal plane, with consistent lateral resolution and minimal field curvature aberration. Our diffractive grating has a maximal angle smaller than ±1.5° for those eight foci, corresponding to a filed curvature sagittal of 9 μm and a field curvature tangential of 8 μm, which are within the depth of field. The F-theta distortion is calculated to be less than 0.01% at ±1.5° (fig. S1A), which leads to consistent beam spot quality at the center and edge (fig. S1, B and C). The diffractive grating features a separation angle of 0.37°, which is the angle between two adjacent beams, and boasts an energy efficiency of ~80% with high uniformity across eight foci. The zero-order diffraction is less than 1% of the incident beam in power, rendering it invisible in the reconstructed image.

To detect the photoacoustic signals, we used two focused ultrasonic transducers, with their acoustic axis aligned with the eight optical foci (Fig. 1). Each transducer has a full width at half maximum of ~0.95 mm along the axis, a central frequency of 34.1 MHz, and a −6-dB bandwidth of 95.2% (fig. S2). The transducer has a focal length of ~7.5 mm with an *f* number of ~1.5. When combined, these transducers provided a relatively uniform detection sensitivity, ensuring sensitivity greater than 50% of the maximum within a 3-mm range. The reflection-mode PUV-PAM, featuring top light illumination and side acoustical detection, is particularly suitable for imaging thick, slide-free specimens. To achieve high imaging speeds, our PUV-PAM system incorporates hybrid scanning methods, including the fast axis by galvo scanning of the eight foci and the slow axis by motor movement (up to 10 mm/s). Consequently, this configuration enhances the imaging speed eightfold over the laser PRR and achieves an imaging speed of 400,000 pixels per second with our 50-kHz laser, which could be further improved with lasers at higher PRRs.

### PUV-PAM of phantom with time-gain compensation

**Fig. 2.**
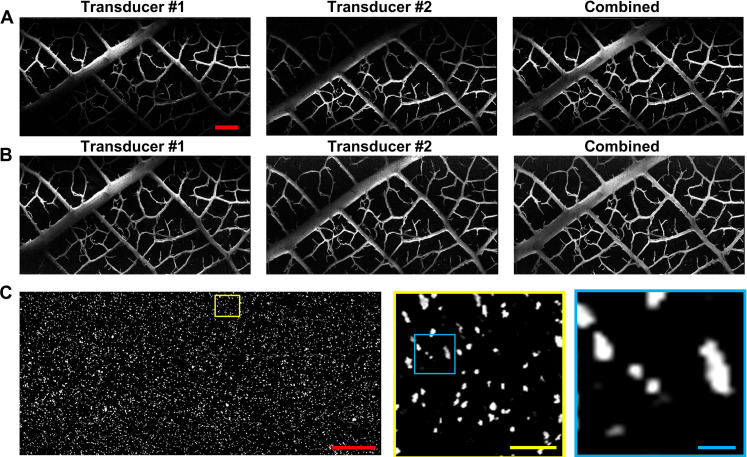
PUV-PAM of leaf skeleton and carbon particles. (**A**) PUV-PAM of a leaf skeleton phantom using single transducers and dual transducers without time-dependent gain compensation (TDGC). (**B**) PUV-PAM of a leaf skeleton phantom using single transducers and dual transducers with TDGC. (**C**) PUV-PAM images of carbon particles using dual transducers with TDGC. Close-up images are extracted from the rectangle areas in corresponding colors. Red scale bars, 500 μm. Yellow scale bar, 50 μm. Blue scale bar, 10 μm.

To test our PUV-PAM, we started by imaging a leaf skeleton phantom embedded in 4% agarose. The PUV-PAM images from a single transducer showed good detail of the leaf skeleton due to the high sensitivity (Fig. 2A). However, images captured from only one transducer exhibited noticeable variations in contrast-to-noise ratio (CNR) within the field of view, particularly displaying higher CNR closer to the focal plane of the acoustical transducer. Using two transducers enhanced the range and uniformity of acoustical detection. Integrating data from both transducers yielded an image with a more uniform CNR across the field of view, although some areas remained comparatively darker (Fig. 2A). To further refine the image quality, we applied time-dependent gain compensation (TDGC), calibrated against the known acoustical detection sensitivity. Post TDGC, even images captured with a single transducer demonstrated a marked enhancement in uniformity (Fig. 2B). It is worth noting, however, that larger structures, like major branches of the leaf skeleton, still presented challenges due to acoustical signal attenuation when propagating to the side. These larger branches exhibited higher signal intensity on the side nearer to the transducer. Using both transducers mitigated this nonuniformity, offering a more realistic representation by capturing signals from two sides (Fig. 2B).

We also imaged carbon particles ranging from 2 to 12 μm in diameter embedded in 4% agarose to representing the size of cell nuclei (Fig. 2C). Close-up examinations of even the edge area within the images showed no distortion and maintained good resolution, identifying the smallest particles around 3 μm (Fig. 2C). The demonstrated capabilities of PUV-PAM, coupled with the TDGC and dual transducers, affirm its suitability for rapid, slide-free photoacoustic histology applications.

### Slide-free and label-free PUV-PAM of fresh tissues

**Fig. 3.**
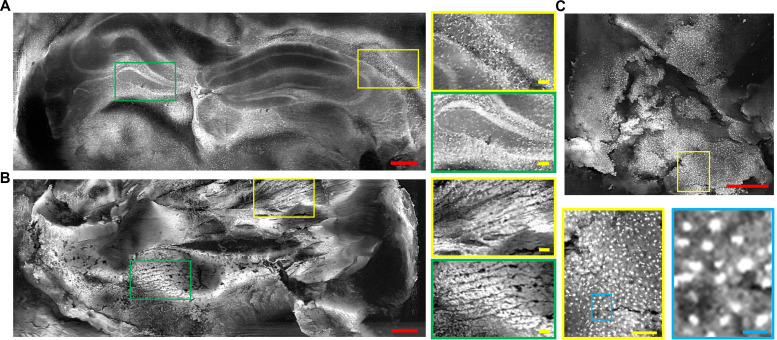
Slide-free PUV-PAM of fresh animal tissues. (**A**) PUV-PAM of a fresh mouse brain cross section and close-up images in representative areas. (**B**) PUV-PAM of a fresh mouse heart cross section and close-up images in representative areas. (**C**) PUV-PAM of a fresh mouse liver and close-up images extracted from selected areas in corresponding colors. Red scale bars, 500 μm. Yellow scale bars, 100 μm. Blue scale bar, 20 μm.

To demonstrate the ability of PUV-PAM to produce histology-like images, we imaged fresh organs harvested from animals. Before imaging, the organs were washed with phosphate-buffered saline to eliminate any residual blood on their surfaces. This was followed by embedding the organs in low-temperature agarose, creating a stable medium for cutting. A vibratome was then used to cut the embedded tissue to expose the cross-sectional area. PUV-PAM imaging of the fresh mouse brain revealed distinct structures such as the cortex, hippocampus, lateral ventricle, and thalamus areas (Fig. 3A). The detailed close-up image provides a clearer view of individual neurons in the hippocampus (Fig. 3A). For comparison, PUV-PAM images reconstructed with only one transducer can be found in fig. S3. PUV-PAM images of the mouse heart provided clear images of cardiomyocytes and the aligned striated muscle fibers indicative of myofibrils (Fig. 3B). However, it should be noted that cutting the fresh and soft organs with a vibratome does not always result in a surface flat enough for high-resolution microscopy. This is especially true for organs with complex structures, such as the heart, where the muscle and ventricles might not be leveled, which could result in some out-of-focus areas in the PUV-PAM image. In the same way, PUV-PAM imaging of the fresh mouse liver showed the liver tissue and cell nuclei with clear details, while zoomed-in views revealed the fine structure of the hepatic cell nucleus (Fig. 3C). We also imaged the fresh mouse cerebellum, mouse kidney, and pig liver with PUV-PAM, as shown in figs. S4 to S6. These results confirm that PUV-PAM is effective for fast slide-free imaging of different tissues.

### *Z*-stack PUV-PAM for slide-free tissues with irregular surfaces

**Fig. 4.**
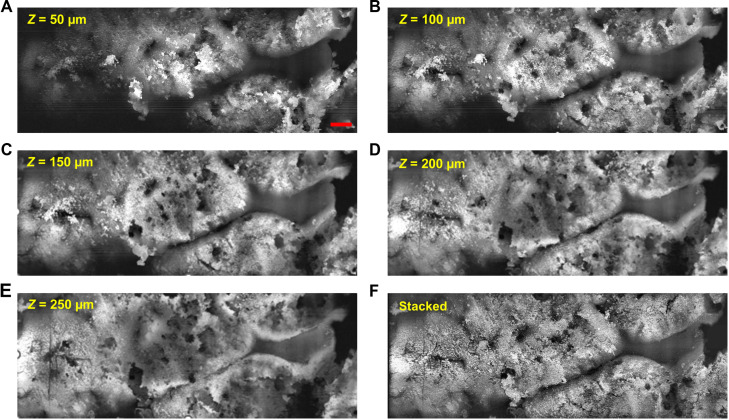
PUV-PAM of fresh slide-free mouse liver. (**A** to **E**) PUV-PAM of fresh mouse liver at representative *z* positions from 50 to 250 μm. (**F**) Maximal projection along the *z* direction from 11 PUV-PAM images acquired within 0 to 250 μm at an interval of 25 μm. Scale bar, 500 μm.

When imaging slide-free tissues that have irregular surfaces, the *z*-stack imaging technique becomes necessary for high-resolution microscopy with a limited depth of field. In our PUV-PAM of fresh mouse liver with non-flat surface, we used *z*-stack imaging at intervals of 25 μm, smaller than the 40-μm depth of focus. This interval ensures that all layered images combined in the stack can capture the whole field of view in high resolution. Representative images at positions of 0 to 250 μm with a 50-μm interval can be found in Fig. 4 (A to E). In those images, it is evident that some areas of the tissue are in sharp focus, while others exhibit out-of-focus blurring at each *z* position. For the stacked image (Fig. 4F), we calculate maximal amplitude projections along the *z* axis from images acquired from different planes. Benefit from the high imaging speed of our system, capturing each image only takes about 60 s for a field of view of 8 mm by 3 mm at 1.3-μm resolution with the scanning step size of 1 μm. Consequently, the entire process of generating *z*-stacked images consisting of 11 positions (i.e., 0 to 250 μm with a 25-μm interval) for slide-free photoacoustic histology was completed within 11 min (movie S1). When compared to our previous contour-scanning UV-PAM techniques (*17*), this method is still five times faster, despite the necessity of scanning at multiple *z* positions. This advancement demonstrates the efficacy of PUV-PAM in handling irregular surfaces of slide-free specimens in a time-effective manner.

### PUV-PAM with needle-shaped beams for extended depth of field

**Fig. 5.**
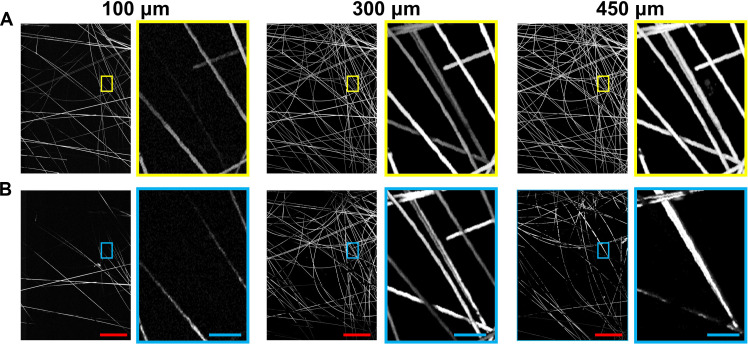
PUV-PAM with an extended depth of field using needle-shaped beams. (**A**) PUV-PAM of a 3D distributed carbon fiber phantom at three *z* positions using needle-shaped beams. (**B**) PUV-PAM of the same phantom at three *z* positions using Gaussian beams. Close-up images are extracted from selected rectangle areas for a more detailed comparison. Red scale bars, 500 μm. Blue scale bars, 50 μm.

To further enhance the imaging speed of the PUV-PAM on specimens with irregular surfaces and avoid *z* scanning, we explored the method of extending the depth of field by combining the diffractive grating and another customized DOE to generate multiple needle-shaped beams. Specifically, the diffractive grating functions as a beam splitter, creating eight focused Gaussian beams via the objective lens, while the other transforms these eight focused Gaussian beams into eight needle-shaped beams. The needle-shaped beam can be considered as multiple foci along *z* direction, with optimization of side lobes, axial uniformity, and energy efficiencies (*18*). The DOE for the needle-shaped beam is designed to extend the depth of field from 40 to 200 μm while preserving a resolution of 1.3 μm and an efficiency of about 15%. The DOE for the needle-shaped beam is placed before the diffractive grating and objective lens. To demonstrate its performance, we imaged a phantom with 3D distributed 6-μm-diameter carbon fibers embedded in 4% agarose. For comparison, the phantom was imaged at various axial positions in an interval of 25 μm using PUV-PAM with both Gaussian beams (movie S2) and needle-shaped beams (movie S3). As demonstrated in Fig. 5, representative PUV-PAM images using needle-shaped beams (Fig. 5A) showed more visible structures compared to PUV-PAM with Gaussian beams (Fig. 5B) at depths of 100, 300, and 450 μm, benefiting from the extended depth of field. The lack of visible features in the Gaussian beam PUV-PAM images is primarily attributed to blurring and signal-to-noise ratio drop due to out of focus. The focal plane for the Gaussian beams is around the *z* position of 300 μm.

Compared to our previous work with needle-shaped beam PAM (*18*), the current PUV-PAM system achieves about a 40-fold increase in imaging speed. This improvement is a result of a higher laser PRR contributing to a fivefold increase (50 kHz as opposed to 10 kHz) and an eightfold acceleration from parallel scanning of eight needle-shaped beams, achieved by the hybrid galvo and motor scanning. The extended depth of field enabled by needle-shaped beams in PUV-PAM holds potential for rapid, slide-free imaging of thick tissues with irregular surfaces, paving the way for more efficient photoacoustic histology applications in clinics.

## DISCUSSION

In this study, we have demonstrated that PAM using a 50-kHz PRR pulsed laser in conjunction with single-element transducers can be expedited through the parallel scanning of multiple beams, where photoacoustic signals from different beams are distinguished on the basis of their time-of-flight information. This process is further aided by a hybrid scanning approach that uses both a galvo mirror for fast scanning of optical beams and a step motor for slow scanning of tissue. Another technical aspect is the implementation of TDGC, which helps in maintaining relatively uniform sensitivity when using two focused single-element ultrasonic transducers positioned on the sides. This approach greatly reduces the need for more costly hardware components, such as high-end lasers, ultrasonic arrays, and multichannel data acquisition systems, ultimately leading to a more cost-effective solution. Moreover, our findings illustrate that incorporating an additional DOE to create needle-shaped beams, used alongside a diffractive grating for beam splitting, allows for the generation of multiple needle-shaped beams for PUV-PAM. This effectively extends the depth of field, which is important for slide-free tissues with uneven surfaces.

While our current UV-PAM system offers substantial advancements, it is important to acknowledge its limitations. One major limitation is the lack of *z* information due to the positioning of the ultrasonic transducers. Because we rely on time-of-flight information to distinguish photoacoustic signals from different beams, our system does not extract optical *z*-axis information as effectively as conventional PAM systems with coaxial alignment of light and acoustic paths. In addition, the side detection of acoustic signals means that a portion of these signals must travel through a certain path length in tissue to reach the detectors. This can slightly reduce the sensitivity of acoustic detection, although PUV-PAM signals primarily originate from superficial tissue layers. Recently, the single-detector method has been introduced to high-speed photoacoustic imaging, potentially accelerating data acquisition in UV-PAM (*26*, *27*). For example, combining multifocal illumination with an acoustic ergodic relay could offer a viable approach for parallel PAM (*28*). However, the acoustical detection sensitivity needs to be improved for UV-PAM owing to the limited pulse energy of UV lasers.

Furthermore, the efficiency of DOEs also affects the overall utilization of laser energy in our UV-PAM system. The diffractive beam splitter, used for multi-focus illumination, has a transmission efficiency of about 80 to 90%. In contrast, the customized DOE designed for creating needle-shaped beams to extend the depth of field has an efficiency ranging between 10 and 30%, depending on its specific design. This leads to a reduction in the available laser energy for multi needle-shaped beam PAM applications. This is particularly relevant in scenarios that require high CNRs with a limited laser power, where optimal energy utilization is crucial for achieving high-quality imaging results. To further improve the DOE efficiency, we would suggest combining the diffractive grating and the DOE for needle-shaped beams, adding antireflective coating, and generating more height levels.

One common challenge in UV-PAM is the relatively poor *z* resolution (i.e., >30 μm), limited by the frequency and bandwidth of the acoustical transducers. This results in a worse sectioning capability compared to conventional formalin-fixed paraffin-embedded (FFPE) slides (i.e., 4 to 7 μm). The current PUV-PAM system also suffered from the insufficient axial resolution due to the side detection, although the limited UV penetration could provide certain sectioning capability (i.e., 50 to 100 μm depending on tissues). To achieve a better axial resolution and sectioning capability like FFPE, nonlinear (*29*), photoimprint (*30*), and Grueneisen relaxation PAM (*31*) can be explored in future PUV-PAM development. Another caveat is the use of agarose embedding in the study for vibratome to create a relatively flat surface in the animal (i.e., mouse) tissue, which takes extra time and may be avoided in clinical settings. For large and thick tissues, it is worth noting that vibratome cutting can work directly on fresh tissues without agarose embedding. In addition, with the enhanced DOF via needle-shaped beams, the requirement of surface flatness can be largely reduced, which may also help avoid the vibratome cutting.

Looking ahead, the implementation of more advanced ultraviolet lasers with increased power and higher PRR has the potential to further enhance the imaging speed. This improvement would be achieved through the generation of more focal spots at a higher PRR. In our existing setup, achieving high CNR in histology-like images requires less than 10 nJ per focal spot. Considering the efficiency of DOE for needle-shaped beams, we are now using a laser pulse energy of ~1 μJ at 50 kHz for PUV-PAM with needle-shaped beams. By upgrading laser power or laser repetition rate, we could potentially boost the imaging speed further with more foci or a higher PRR, reaching 5 to 10 million pixels per second. To further improve the accessibility of photoacoustic histology for clinical applications, the cost of UV light source will also need to be lowered. In addition, optimizing the DOE efficiency for needle-shaped beams could lead to better integration with diffractive gratings, which would enable faster intraoperative slide-free histology imaging using multiple need-shaped beams. These advancements aim not only to improve the performance of PUV-PAM systems but also to broaden their use in clinical settings.

## MATERIALS AND METHODS

### Details of reflection-mode PUV-PAM

The reflection-mode PUV-PAM system uses a 266-nm nanosecond pulsed laser with a PRR up to 50 kHz (QL266-010-O, CrystaLaser). The laser beam is first expanded using a combination of a plano-concave lens and a plano-convex lens, subsequently directing the beam toward a 2D galvo scanning mirror (GVS412, Thorlabs). After the galvo mirror, the beam passes through a 4-f system composed of two lenses, which relays it to the custom-designed F-theta objective lens (Avantier Inc.). A photodiode was used to compensate for laser energy fluctuation. Two DOEs are positioned in front of the objective lens. The first DOE is engineered to produce a needle-shaped beam, extending the depth of focus. The second DOE functions as a diffractive beam splitter, creating eight parallel foci aligned linearly with an inter-focal spacing of 375 μm. When combined, these DOEs generate eight simultaneous needle-shaped beams. Without the first DOE for needle-shaped beams, it generates eight focused Gaussian beams.

Detection of photoacoustic signals generated by the optical foci is achieved using two custom ultrasonic transducers arranged face to face, with their longitudinal axes aligned with the eight optical foci. The acoustical foci of these transducers are deliberately aligned at a distance from each other, ensuring relatively uniform sensitivity across all optical foci when combining two transducers. The separation of the photoacoustic signals from individual focus is facilitated through time-of-flight differentiation, with a temporal discrepancy of 250 ns, corresponding to a distance of 375 μm at a speed of 1500 m/s. The reflection-mode PUV-PAM fast optical scanning is performed along the axis of the eight optical foci using the galvo mirror, enabling simultaneous scanning of all eight beams. The slow axis scanning of tissues is implemented by a step motor (PLS-85, PI) moving perpendicular to the fast axis.

The photoacoustic signals are amplified using two low-noise amplifiers (ZFL-500LN+, Mini-Circuits) and are digitized at a 500-MHz sampling rate by a high-speed data acquisition card (ATS 9350, AlazarTech). During scanning, data are transferred to a computer equipped with a nonvolatile memory express M.2 solid-state drive, ensuring a data transfer speed of 400 MB/s. The synchronization of laser pulses, galvo mirror scanning, motor movement, and data acquisition is achieved through a multifunction input/output (I/O) device (PCIE-6323, National Instruments) and customized LabVIEW program.

### Fresh organ preparation

Fresh organs were harvested from C57BL male mice (Envigo), followed by a thorough wash with phosphate-buffered saline to remove any surface blood. These washed organs were then submerged in 4% low–melting point agarose solution at a temperature of 37° to ensure proper embedding. The agarose containing the organs was cooled in a refrigerator set to 4°C for 15 min, solidifying it into robust gel blocks. Once solidified, the agarose-embedded organ blocks were securely mounted onto a sample holder via glue. The sample holder was mounted on a 3D motorized stage for positioning and scanning. The samples were then transferred to a vibratome via the motorized stage, which sliced the fresh tissue surfaces to reveal cross sections for PAM imaging. After preparation, the tissues were positioned in the designated area for imaging. The sample holder, with the embedded organ tissue, was immersed in a water tank during imaging for acoustical coupling. The histology-like PAM images were captured using hybrid galvo scanning of optical beams and motor scanning of tissues.

### Phantom preparation

The preparation of the carbon particle and carbon fiber phantoms involved embedding ~6-μm-diameter carbon fibers and 2- to 12-μm-diameter carbon particles (484164, MilliporeSigma) within a 4% agarose block (A9045, MilliporeSigma). The process began with heating water to a temperature range of 70° to 80° to ensure the complete dissolution of the agarose powder. Once fully dissolved, the 4% agarose solution was cooled in a 32° water bath, which prevents the agarose from solidifying too quickly while maintaining a viscosity conducive for evenly distributing carbon fibers and carbon particles in a 3D arrangement. Then, the solidification of the agarose phantom was then expedited by refrigerating it at 4° for 15 min. In the preparation of the leaf phantom, a leaf skeleton was first coated with black ink, enhancing photoacoustic contrast at the 266-nm wavelength. This inked leaf skeleton was placed on a preformed 4% blank agarose block. To embed the leaf within the agarose, more agarose solution at ~40° was gently poured over it. This additional layer of agarose ensured that the leaf skeleton was fully encapsulated and the structural flatness was maintained. After the complete solidification of the agarose, the specimens were carefully positioned in the sample holder for subsequent PAM imaging.

### Data and image processing

To separate the photoacoustic signals from the eight beams, we divided the time domain signals received by the two ultrasonic transducers into eight specific time windows, based on the location of the beam spots relative to the transducers. We then calculated the amplitudes of the photoacoustic signals within these time windows after applying a Hilbert transform. The calculated amplitudes were then processed with the TDGC method, based on pre-calibrated transducer sensitivity along the acoustical axis. Then, these amplitudes from both transducers were combined to form eight total amplitudes. This approach was chosen because the focus of each ultrasonic transducer was aligned at the midpoint between the second and third beam spots to the transducer, ensuring consistent detection sensitivity along all eight beams. For reconstructing the 2D PAM images of whole field of view, we computed the maximum amplitude projection images from each focus. These projections were stitched together to create a larger field of view. To improve the accuracy of this stitching, each subsection overlapped by 5 pixels in the direction of the galvo scanning. For the *z*-stacked PAM image, we calculated the MAP images of all *z* positions first and took maximal values along the *z* direction from the calculated MAP images.
