# Supplementary material for: Optical-resolution parallel ultraviolet photoacoustic microscopy for slide-free histology
Source: Sci Adv. 2024 Dec 11;10(50):eado0518. doi: 10.1126/sciadv.ado0518 (PMC11633733; doi:10.1126/sciadv.ado0518)
Supplement: Supplementary file 1 — Figs. S1 to S6 Legends for movies S1 to S3 [file sciadv.ado0518_sm.pdf]

Science Advances
AAAS

Supplementary Materials for

Optical-resolution parallel ultraviolet photoacoustic microscopy for slide-free histology

Rui Cao *et al.*

Corresponding author: Lihong V. Wang, lvw@caltech.edu

Sci. Adv. **10**, eado0518 (2024)
DOI: 10.1126/sciadv.ado0518

The PDF file includes:

Figs. S1 to S6
Legends for movies S1 to S3

Other Supplementary Material for this manuscript includes the following:

Movies S1 to S3

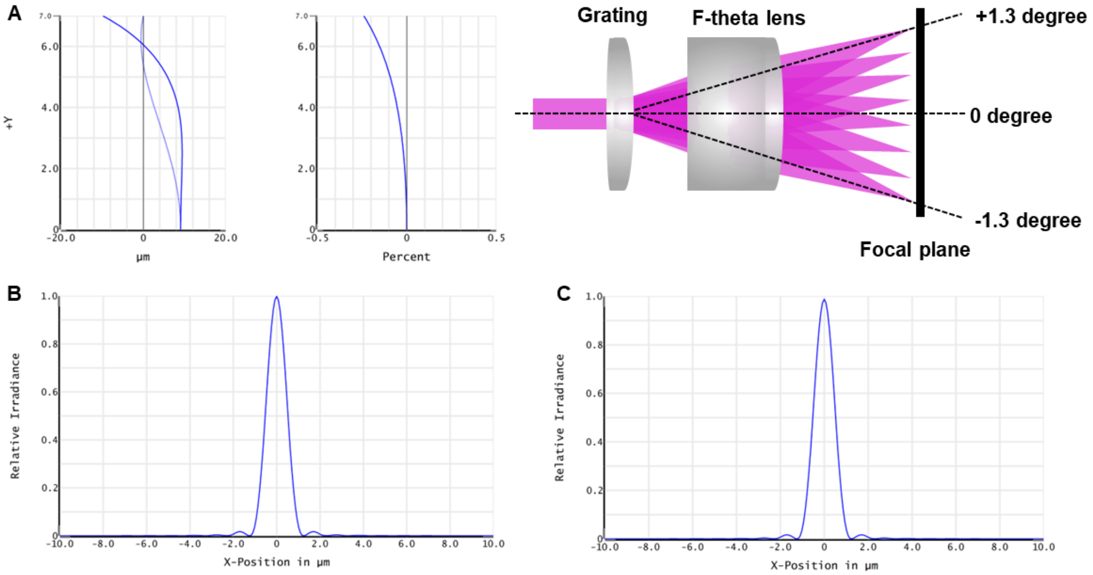

Figure S1. **Characterization of the customized F-theta lens.**
(A) Field curvature and F-theta distortion of the customized F-theta lens and the schematic of the F-theta lens with the 1D diffractive grating. **(B)** Cross-section PSF of the focused spot at 0 degree. **(C)** Cross-section PSF of the focused spot at 7 degrees.

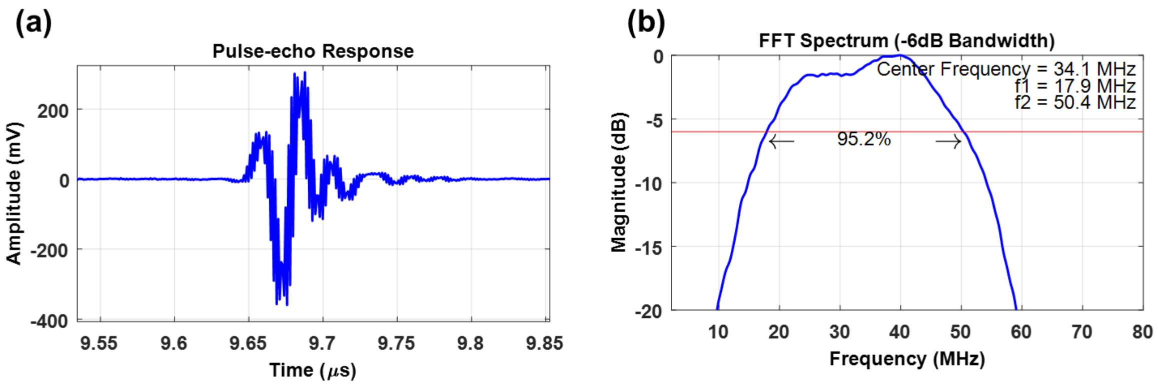

Figure S2. Characterization of the customized ultrasonic transducer.
(A) Pulse-echo responses of the customized transducer. **(B)** Transducer frequency response from the pulse-echo measurement.

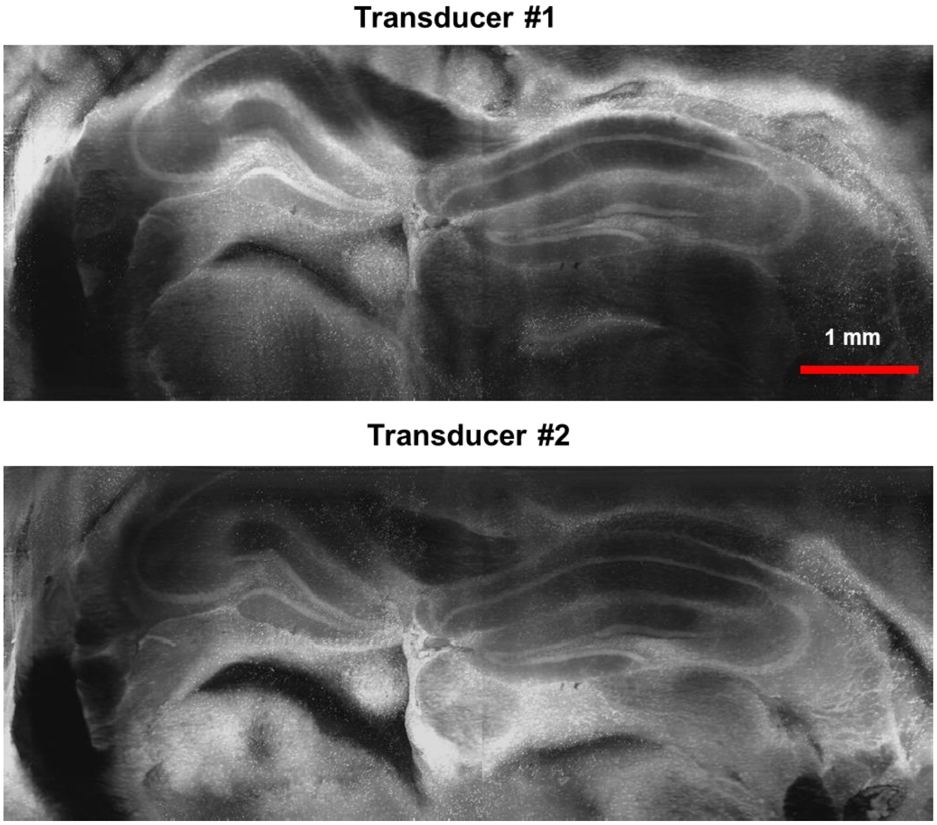

Figure S3. PUV-PAM images of the mouse brain cross section reconstructed with only one transducer.

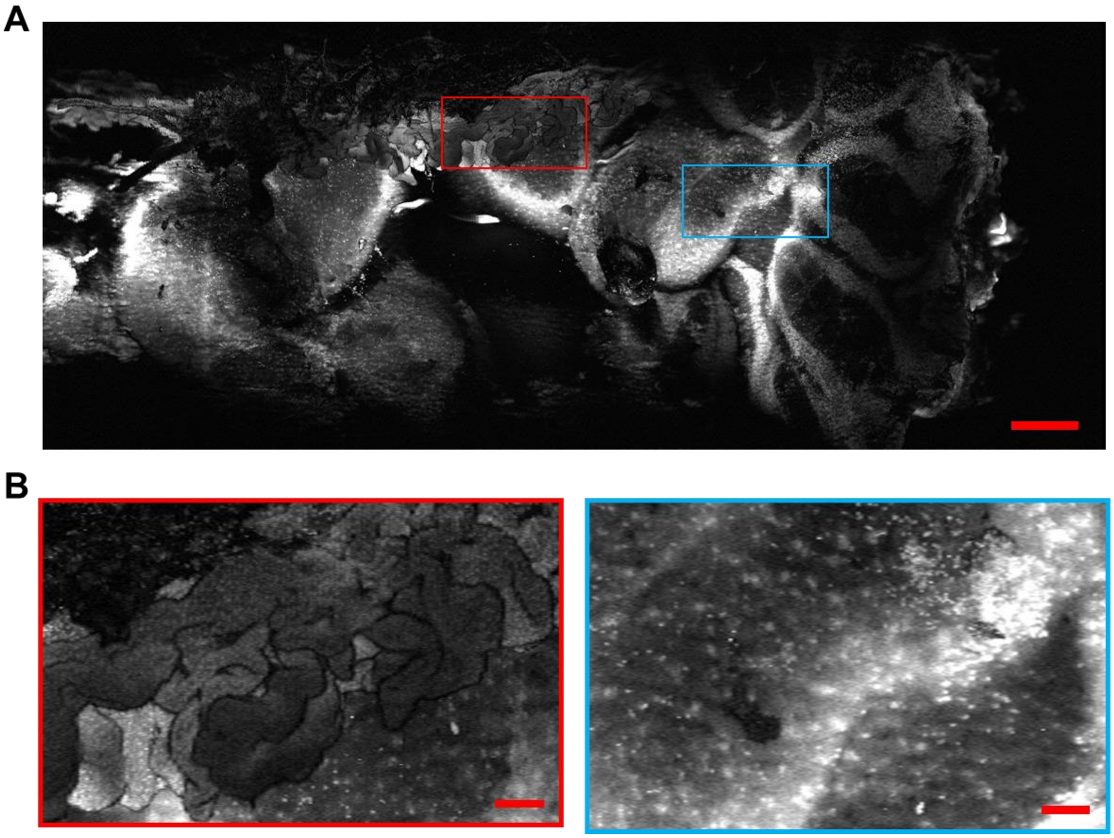

Figure S4. **PUV-PAM of slide-free fresh mouse cerebellum.**
(A) PUV-PAM image of fresh mouse cerebellum showing the large field of view. Scale bar, 500 µm. **(B)** Close-up images from the selected areas showing more detailed structures. Scale bar, 100 µm.

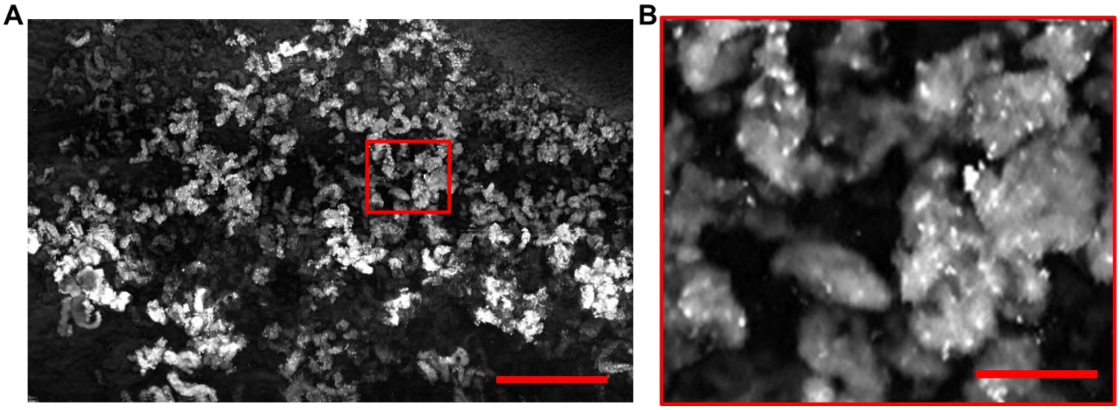

Figure S5. **PUV-PAM of slide-free fresh mouse kidney**
(A) PUV-PAM image of fresh mouse kidney showing the large field of view. Scale bar, 500 µm.
(B) Close-up image from the selected area. Scale bar, 100 µm.

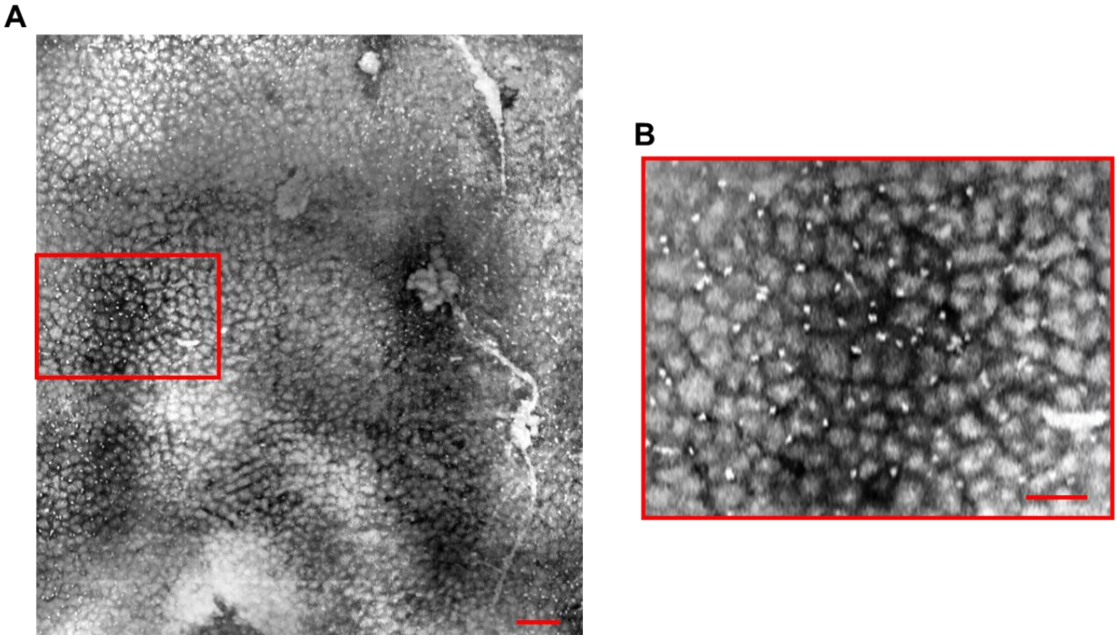

Figure S6. **PUV-PAM image of slide-free fresh pig liver.**
(A) PUV-PAM image of fresh pig liver showing the large field of view. Scale bar, 100 µm. **(B)** Close-up image from the selected area. Scale bar, 50 µm.

Movie S1.
Z-stack PUV-PAM of slide-free fresh mouse liver at different z positions.

Movie S2.
PUV-PAM of carbon fiber phantoms at different z positions via Gaussian beams.

Movie S3.
PUV-PAM of carbon fiber phantoms at different z positions via needle-shaped beams.
